# Distribution bias and biochemical characterization of *TOP1MT* single nucleotide variants

**DOI:** 10.1038/s41598-017-09258-2

**Published:** 2017-08-17

**Authors:** Hongliang Zhang, Yeonee Seol, Keli Agama, Keir C. Neuman, Yves Pommier

**Affiliations:** 1 Laboratory of Molecular Pharmacology, Developmental Therapeutics Branch, Center for Cancer Research, NCI, National Institutes of Health, Bethesda, MD 20892 USA; 2 Laboratory of Single Molecule Biophysics, NHLBI, National Institutes of Health, Bethesda, MD 20892 USA

## Abstract

Mitochondrial topoisomerase I (TOP1MT) is a type IB topoisomerase encoded in the nucleus of vertebrate cells. In contrast to the other five human topoisomerases, *TOP1MT* possesses two high frequency single nucleotide variants (SNVs), rs11544484 (V256I, Minor Allele Frequency = 0.27) and rs2293925 (R525W, MAF = 0.45), which tend to be mutually exclusive across different human ethnic groups and even more clearly in a cohort of 129 US patients with breast cancer and in the NCI-60 cancer cell lines. We expressed these two TOP1MT variants and the double-variant (V256I-R525W) as recombinant proteins, as well as a less common variant E168G (rs200673353, MAF = 0.001), and studied their biochemical properties by magnetic tweezers-based supercoil relaxation and classical DNA relaxation assays. Variants showed reduced DNA relaxation activities, especially the V256I variant towards positively supercoiled DNA. We also found that the V256I variant was enriched to MAF = 0.64 in NCI-60 lung carcinoma cell lines, whereas the TOP1MT R525W was enriched to MAF = 0.65 in the NCI-60 melanoma cell lines. Moreover, *TOP1MT* expression correlated with the 256 variants in the NCI-60 lung carcinoma cell lines, valine with high expression and isoleucine with low expression. Our results are discussed in the context of evolution between the nuclear and mitochondrial topoisomerases and potential cancer predisposition.

## Introduction

The occurrence of different forms of an allele in a gene defines genetic polymorphism. Single Nucleotide Polymorphism (SNP) also known as single nucleotide variants (SNVs) refers to single base differences in the genomic DNA. Most known SNPs are not associated with genetic disorders. However, some SNPs that occur within or near coding regions are associated with increased risk for developing certain diseases, including predisposition to cancer, and susceptibility to various environmental conditions.

Mitochondria are essential organelles that provide nucleotides for the nuclear genome and are key for cellular metabolism and apoptosis. The importance of mitochondria is underscored by the increasing number of diseases associated with mitochondrial malfunction, particularly muscular and neurological diseases owing to the high mitochondrial content of myocytes and neurons^1^. Mitochondria contain their own DNA (mtDNA; chromosome M), which encodes a small (13 genes) but essential subset of genes for oxidative phosphorylation and cellular energy generation in the form of ATP. In every mitochondrion, mtDNA consists of multiple copies of a highly conserved circular genome (16,569 base pairs in humans) packaged in nucleoids in which the mtDNA is not free to swivel as it is attached to the inner mitochondrial membrane. In addition, because mtDNA is highly transcribed from divergent promoters, and needs to be replicated as cells divide and adjust their energy requirements, supercoiled and interlinked mtDNA circles are prevalent, requiring the action of topoisomerases^2–4^. DNA topoisomerases, by transiently cutting and re-ligating the DNA backbone, maintain proper DNA supercoiling density and resolve DNA crossovers (catenanes and knots), which explain their requirement for DNA replication, RNA transcription, and protein translation. Vertebrate cells encode six topoisomerases: four type I: TOP1MT, TOP1, TOP3A and TOP3B and two type II: TOP2A and TOP2B. The type I topoisomerases cleave only one strand of DNA (and RNA for TOP3B) to dissipate supercoiling and minimize R-loops and D-loops (and RNA knots for TOP3B)^5^. In contrast, type II topoisomerases cleave both strands of duplex DNA to pass another duplex thereby changing DNA supercoiling and resolving DNA knots and catenanes^4, 6, 7^.

Mitochondrial DNA topoisomerase I (TOP1MT) like the other mtDNA enzymes is encoded by the nuclear genome and exclusively localizes to mitochondria via a mitochondrial targeting sequence at its N-terminus^2^. TOP1MT acts throughout the mitochondrial genome with preferred association with the regulatory regions of mtDNA (non-coding regions containing the promoters for both strands in addition to replication D-loops and replication origins)^3^. Deletion of TOP1MT leads to increased negative supercoiling of mtDNA^8^, and TOP1MT plays a critical role in mtDNA expansion following liver regeneration and mtDNA depletion^9^. It also maintains mtDNA and mitochondrial homeostasis in cardiomyocytes in adaptive response to doxorubicin-induced cardiotoxicity^8^.

TOP1MT is unique among the topoisomerases because of the presence of two frequent SNV alleles in the normal population (V256I and R525W) (see Table 1), which tend to be mutually exclusive in different ethnic group. Also, the V256I SNV, which is more frequent in Africans compared to Asians (see Table 1), corresponds to the conserved isoleucine residue of (nuclear) TOP1, which has previously been shown to be toxic when expressed in mitochondria^10^. Using our previously established single-molecule magnetic tweezer-based techniques^11, 12^ and classical DNA relaxation assays with negatively and positively supercoiled DNA substrate^13, 14^, we tested whether the biochemical activity of the V256I and R525W single variants differed from the double variant V256I-R525W as well as from the activity of a less frequent SNV allele E168G and “wild-type” TOP1MT. Our goal was to determine whether those SNVs were biochemically different from “wild-type” TOP1MT and if so, whether this could explain their distribution bias.

**Table 1:** Minor Allele Frequency (MAF) variants for *TOP1MT*.

	V256I	R525W	Source*
	rs11544484	rs2293925	
Overall	0.27	0.45	1000 genome
Overall	0.27	0.54	
Africans	0.48	0.12	
Europeans	0.29	0.49	ExAC
Latinos	0.16	0.74	
South Asians	0.22	0.68	
East Asians	0.07	0.74	
Africans	0.54	0.00	NCBI
Caucasians	0.30	0.45	
Asians	0.07	0.76	

^*^Source:

1000 genomes: http://browser.1000genomes.org.

ExAc: http://exac.broadinstitute.org.

NCBI: https://www.ncbi.nlm.nih.gov.

## Results

### Two high frequency TOP1MT variant alleles V256I and R525W

**Figure 1:**
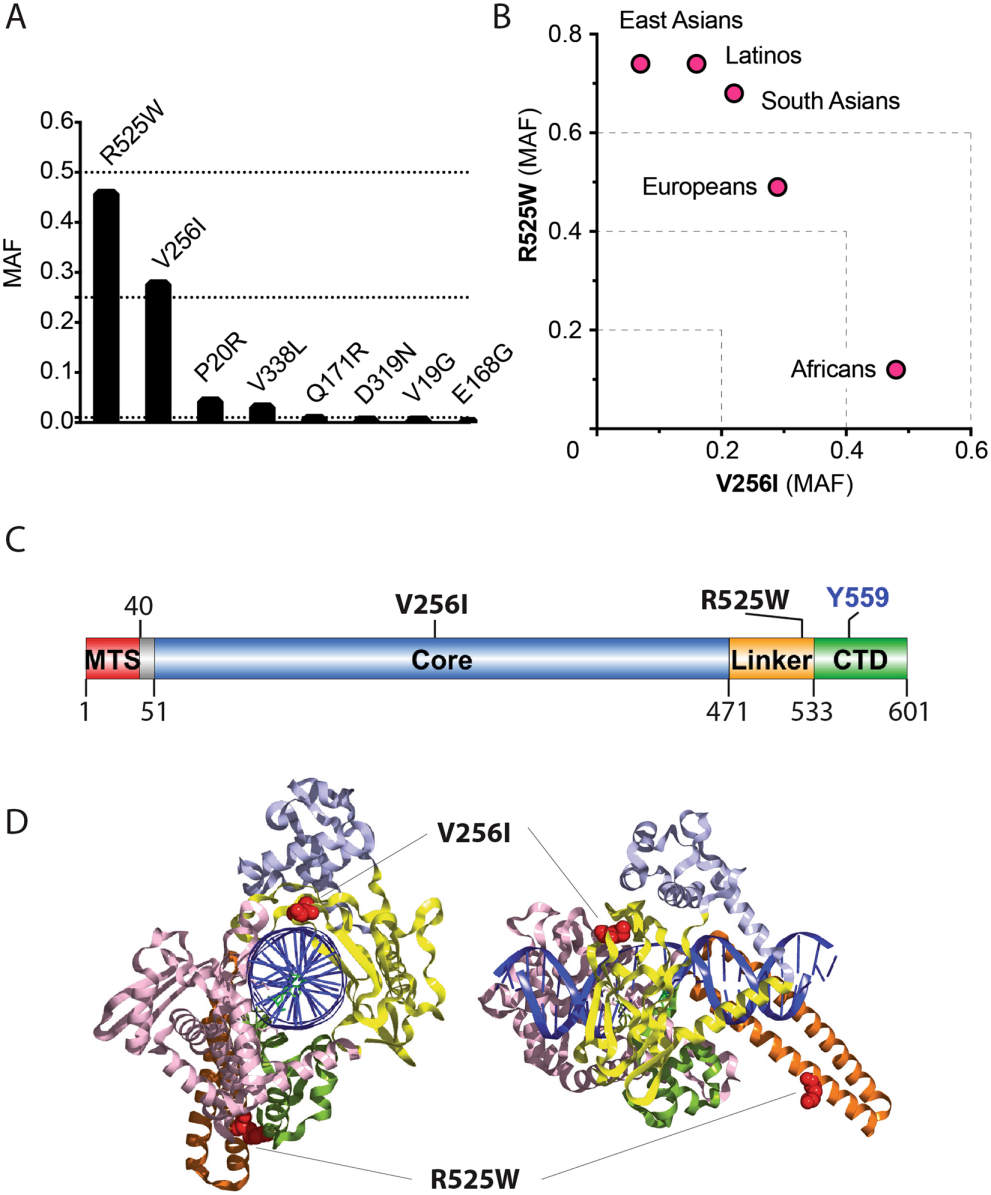
TOP1MT V256I and R525W variants. (**A**) Frequency of the TOP1MT variants in the overall human populations. (**B**) Relative distribution of the V256I and R525W variants across human ethnic groups. (**C**) Positions of the two variants relative to TOP1MT functional and structural domains in linear format. MTS: Mitochondrial Targeting Signal; Core: the core domain; Linker: the linker domain; CTD: C-Terminal Domain. (**D**) representation of the positions of the two variants based on the crystal structure of TOP1^31^ (PDB ID: 1K4T).

Examination of open genomic databases revealed that *TOP1MT* has two high frequency variant alleles. The rs11544484 allele is a Valine (GTT) to Isoleucine (ATT) missense variant at amino acid 256 (V256I). The rs2293925 allele is an aRginine (CGG) to tryptophan (W) (TGG) missense variant at amino acid 525 (R525W). Both variants display higher than 0.25 minor allele frequencies (MAF), the frequency at which the second most frequent allele occurs, indicating that more than 25% of the global human population possess one of these variants (Table 1; Fig. 1A). Overall MAF was 0.27 for the V256I variant and around 0.5 for the R525W variant, putting the W525 variant close to the R525 reference allele. Examination of the MAF across different human populations showed a strong bias with enrichment of the V256I variant in Africans (MAF ≈ 0.5) and of the R525W variant in Asians and Latinos (MAF ≈ 0.75) (Fig. 1B, Table 1).

**Figure 2:**
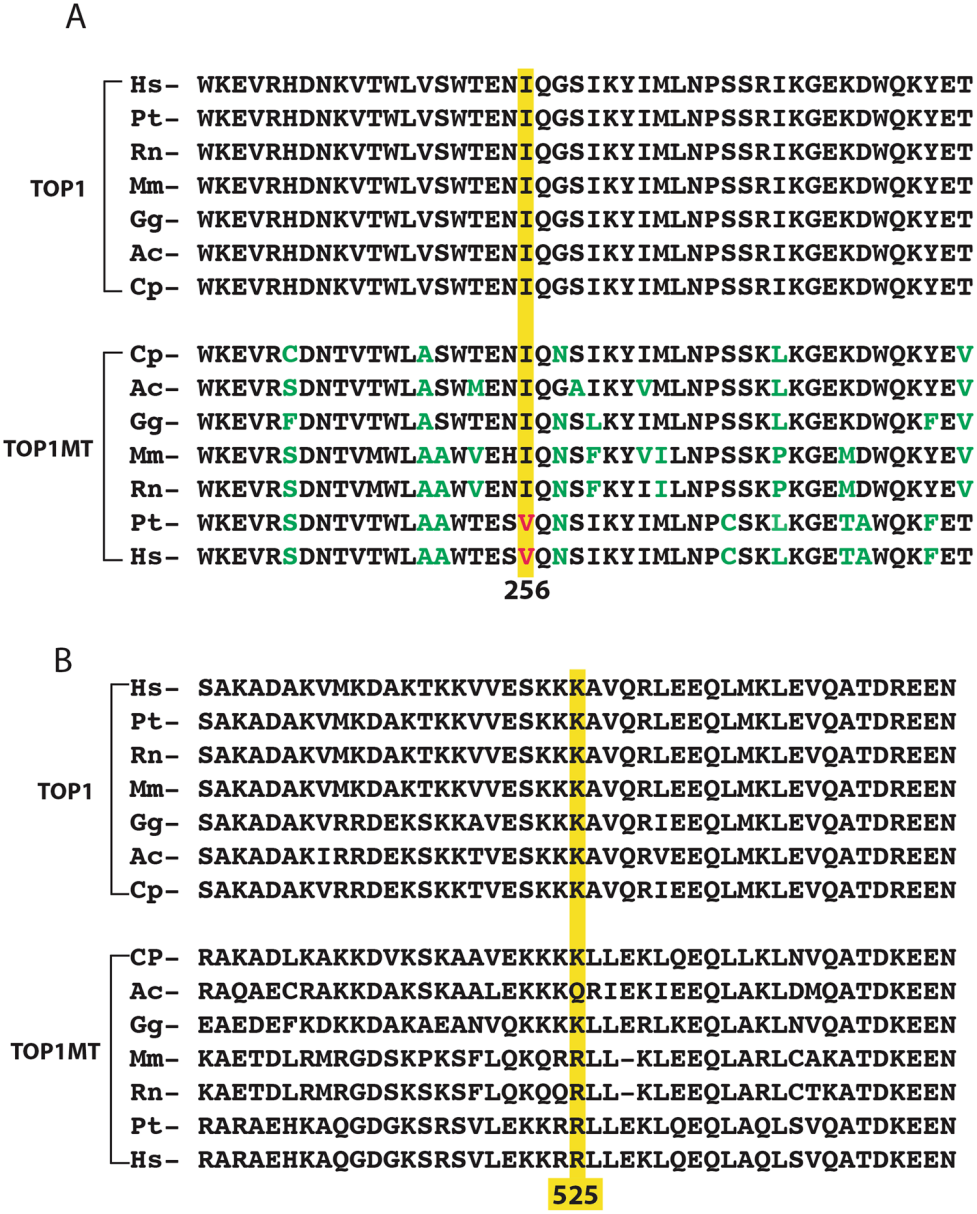
The V256I and R525W variants correspond to highly conserved regions of TOP1MT across species. (**A**) Alignment of the TOP1 and TOP1MT sequences corresponding to the V256I site. (**B**) Alignment of the TOP1 and TOP1MT sequences corresponding to the R525W site. Hs, *Homo sapiens*; Pt, *Pan troglodytes*; Rn, *Rattus norvegicus*; Mm, *Mus musculus*; Gg, *Gallus gallus*; Ac, *Anolis carolinensis*; Cp, *Chrysemys picta*.

The TOP1MT 256 residue is in a highly conserved region of the catalytic core domain of TOP1MT (Figs 1C–D and 2A)^15^. Notably, all reference nuclear TOP1 enzymes across species bear the isoleucine variant at this position (Fig. 2A). The TOP1MT 525 residue is in the linker domain, between the catalytic core and the C-terminal domain containing the catalytic tyrosine of TOP1MT (Fig. 1C–D). This domain is less conserved than the core domains between the nuclear (TOP1) and mitochondrial (TOP1MT) enzymes (Fig. 2C and Supplemental Fig. S1)^15, 16^.

### Distribution bias between the TOP1MT variant alleles V256I and R525W

To further establish whether the two SNP variant alleles V256I and R525W were distributed independently from each other, we analyzed a cohort of 129 patients and determined for individual patient whether each of the two *TOP1MT* alleles (V256I and R525W) was homozygous or heterozygous and wether they coexisted in the same samples. The results shown in Table 2 demonstrate that the distribution of the two alleles was not independent. None of the patients homozygous for the V256I or the R525W allele expressed the other allele (p < 0.001). Similar results were observed by exome sequencing of the lung and melanoma cancer cell lines of the NCI-60^17, 18^ (Table 3) (https://discover.nci.nih.gov/cellminer/). These analyses demonstrate the presence of a strong distribution bias against the double-mutant V256I-R525W in human samples.

**Table 2:** Linkage of the *TOP1MT* 256 and 525 alleles.

	V256 homozygous	256 heterozygous	I256 homozygous	total
R525 homozygous	11	25	28	64
525 heterozygous	23	33	0	56
W525 homozygous	9	0	0	9
total	43	58	28	129

^*^Source.

129 samples from women with breast cancers were used to genotype *TOP1MT* variants for both alleles at positions 256 (V or/and I) and 525 (R or/and W).

Chi-square p value = 0.0005.

**Table 3:** Tissue-specific enrichment of the TOP1MT SNP variants in the NCI-60.

Cell line	Variant (dbSNP id)	
	V256I	R525W
	rs11544484	rs2293925
LC:A549	42	0
LC:EKVX	97	0
LC:HOP_62	100	0
LC:HOP_92	0	0
LC:NCI_H226	100	0
LC:NCI_H23	0	0
LC:NCI_H322M	100	0
LC:NCI_H460	100	0
LC:NCI_H522	33	80
ME:LOXIMVI	0	0
ME:MALME_3 M	19	100
ME:M14	0	100
ME:SK_MEL_2	100	0
ME:SK_MEL_28	0	100
ME:SK_MEL_5	0	100
ME:UACC_257	31	0
ME:UACC_62	0	55
ME:MDA_MB_435	0	100
ME:MDA_N	0	96

Numbers in both columns refer to the percentage of reads with that particular variant.

### Biochemical characterization of the V256I, R525W and E168G TOP1MT variants

To determine if SNP variants have functional consequences on the catalytic activity of TOP1MT, we expressed and purified the TOP1MT SNP variants in parallel with wild type (WT) TOP1MT as recombinant proteins. We also made the V256I-R525W double-variant to determine if its biochemical properties could explain why it tends to be excluded in normal human populations (see above). In our SNP search, we found a rs200673353 allele, corresponding to a E168G variant, which is at a conserved position across the TOP1 and TOP1MT enzymes (Fig. S1). Its potential deleterious nature was supported by its very low penetrance (MAF of 0.001; Fig. 1A). We also expressed the E168G variant and studied its biochemical properties.

**Figure 3:**
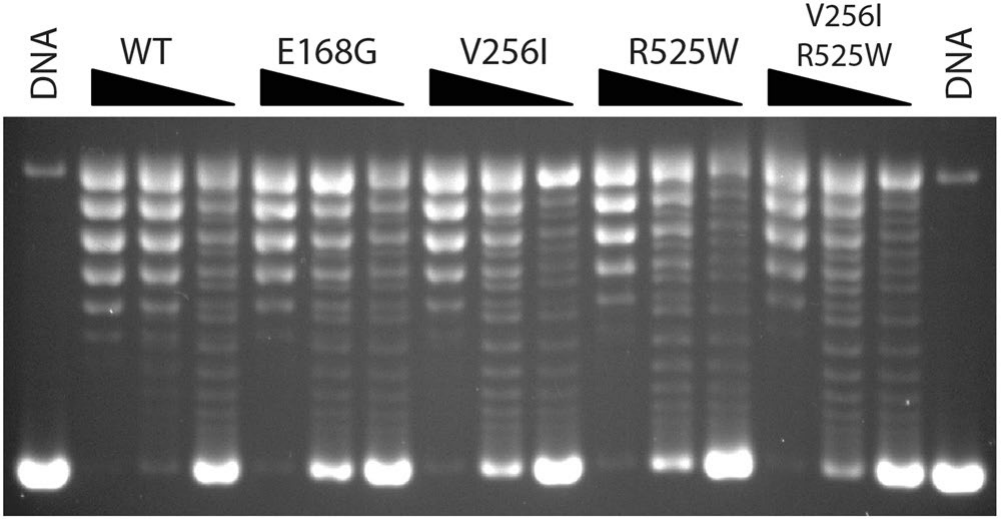
Relaxation activity of TOP1MT wild type and its variants assayed with negatively supercoiled pBR322 plasmid DNA. Equal concentrations (2 ng/μl) of each enzyme were used for a dilution series of 1:1, 1:3 and 1:9. A representative gel was shown.

TOP1 relaxation assays with negatively supercoiled native DNA (Sc−), showed that all the variants were active. However, they exhibited slight but consistently reduced DNA relaxation activities compared to WT TOP1MT (Fig. 3). This suggested that the variants might be less effective than WT TOP1MT in their nicking-closing activity. To test this possibility further, we performed DNA cleavage assays with the canonical TOP1 inhibitor camptothecin (CPT). CPT is an interfacial inhibitor that blocks the DNA ligation (closing) step of the nicking-closing reaction of TOP1^19^ and TOP1MT^2, 12^. All four variants (V256I, R525W, E168G and V256I-R525W) showed CPT-induced cleavage sites at the same sequences as the WT enzyme. However, the efficiency of CPT stabilized cleavage was consistently less than WT TOP1MT (Fig. S2), likely due to altered nicking-closing activity for the variants.

### Single molecule DNA supercoil assays

**Figure 4:**
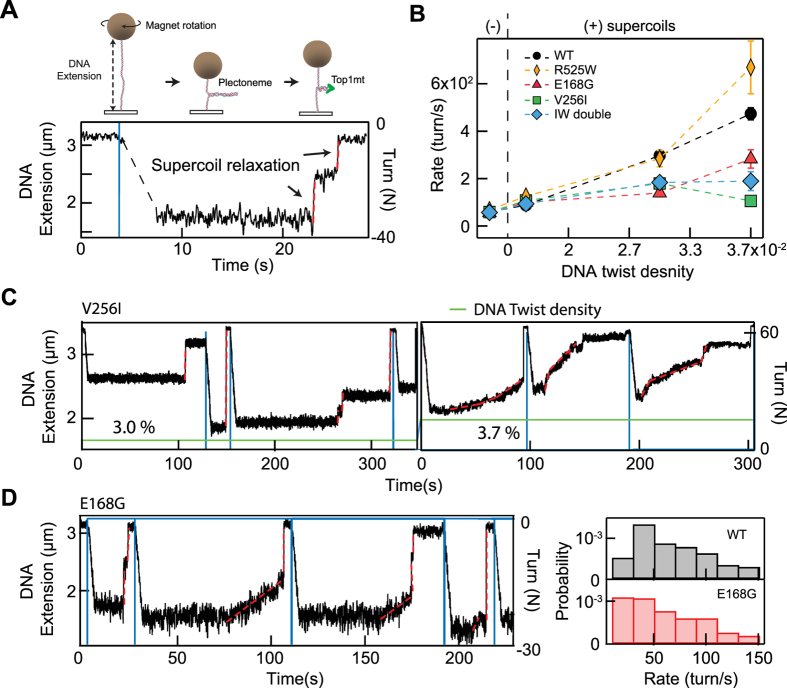
Single-molecule assay to characterize DNA supercoil relaxation activity of WT TOP1MT and three TOP1MT variants. (**A**) Schematic of single-molecule DNA supercoil generation and measurement of TOP1 relaxation using magnetic tweezers (not to scale). The DNA extension decreases as supercoils are introduced in the DNA molecule by rotating DNA tethered magnetic bead with the external magnets (see methods). The DNA extension increases as TOP1 relaxes the supercoiled DNA molecule. Individual TOP1 supercoil relaxation events (highlighted with dashed red lines) are analyzed to extract the rate of supercoil relaxation and the number of supercoils relaxed per cleavage-religation cycle. (**B**) The supercoil relaxation rate for WT and three TOP1MT variants as a function of the imposed twist on the DNA, proportional to the torque applied on the DNA. (**C**) Single-molecule relaxation measurements illustrating decreased relaxation activity of V256I TOP1MT variant under high positive DNA twist density (3.7%). The DNA extension is plotted as a function of time (black line, left axis), the introduction of supercoils is indicated by the blue lines (right axis), the twist density is reported as a percentage and indicated by the green line. Note the variation in relaxation rate during individual relaxation events (e.g., at ~100 s on the right panel) and the increased prevalence of slow relaxation events at higher (3.7%) twist density (right panel) compared to the lower (3.0%) twist density (left panel) (**D**) Example of random switching between slow and WT relaxation rates for E168G TOP1MT (left panel). The distributions of relaxation rates indicate a higher probability of slow relaxation rate events (<20 turn/s; marked as shade) for the E168G variant compared to WT Top1mt (right panel).

To expand our examination of the biochemical activities of the TOP1MT variants, we performed magnetic tweezers-based single-molecule supercoil relaxation assays that specifically probe the supercoil relaxation rate for different levels of DNA twist density (or torque)^11, 12, 20, 21^ (Fig. 4A).

We found that all TOP1MT variants, except R525W, showed an overall slower relaxation rate than WT TOP1MT, particularly at higher positive twist density, indicating that SNPs adversely affect TOP1MT catalytic activity (Fig. 4B). In particular, the relaxation rate of the V256I variant slowed down dramatically as DNA twist density increased (Fig. 4B and C). Interestingly, the double V256I-R525W mutant also exhibited a decrease in relaxation rate with increasing DNA twist density (force) but to a lesser degree than the V256I mutant. It is possible that the R525W mutation partially compensates for the effects of the V256I mutation since the R525W mutant showed comparable, even slightly enhanced, relaxation rates compared with the WT enzyme. The adverse effects of the E168G mutation on catalytic activity were apparent at low negative DNA twist density (−0.004), whereas the catalytic activity of the other variants were comparable with WT at this twist density. Analysis of individual relaxation events of the E168G variant on negatively supercoiled DNA reveal random switching between slow relaxation events and fast (comparable to WT) relaxation events (Fig. 4D). This variability is reflected in the increase in the probability of slow (<20 turns/s) relaxation rates for E168G compared to that of WT (Fig. 4D).

### Defective efficiency of the V256I TOP1MT variant in relaxing positive supercoiling

**Figure 5:**
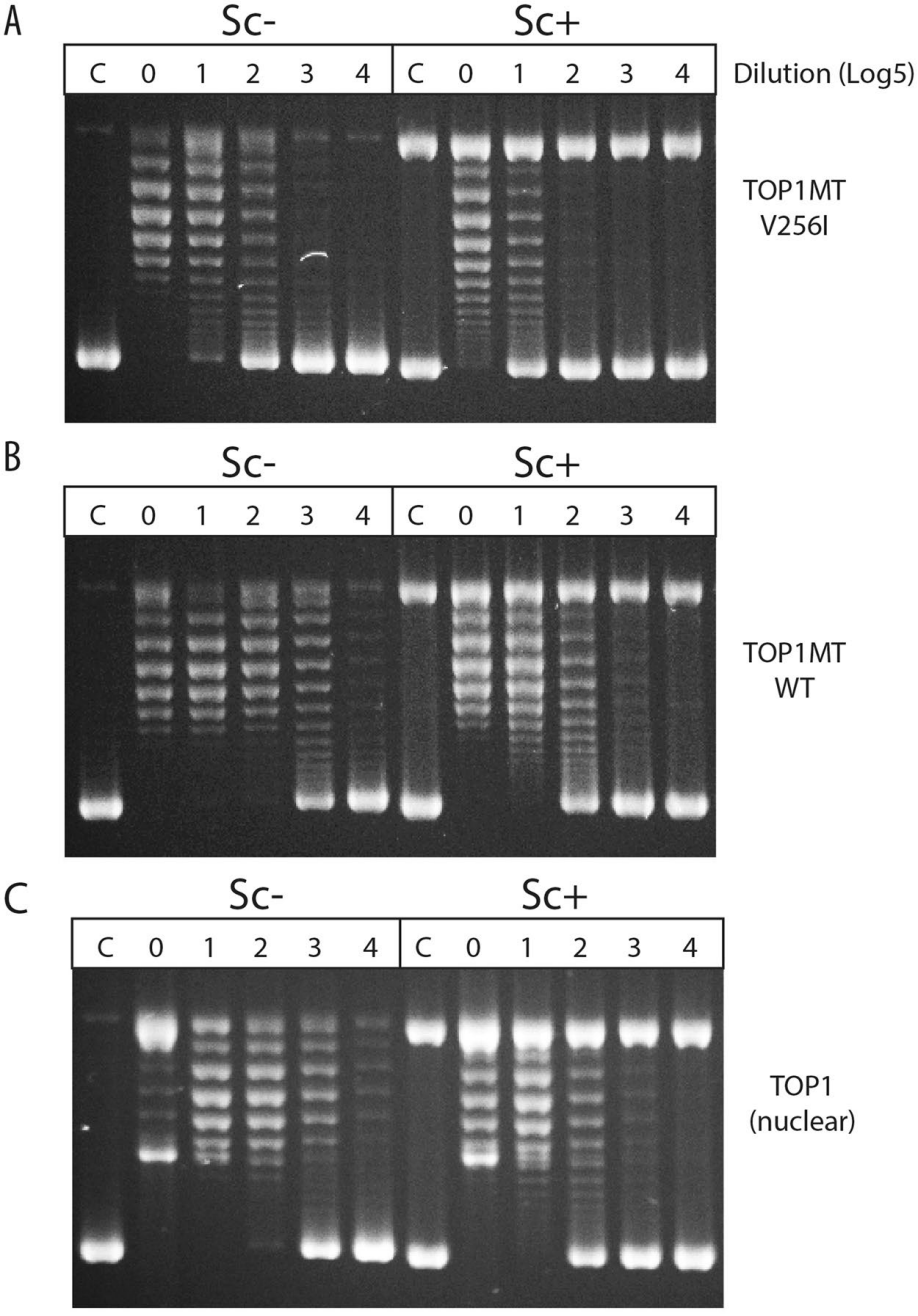
Relaxation assay of TOP1MT, TOP1MT V256I variant, and TOP1 on negatively and positively supercoiled pBR322 DNA. Sc−: Negatively supercoiled pBR322 plasmid DNA; Sc+ : positively supercoiled pBR322 plasmid DNA; C, control, no enzyme added; the numbers on top of the figures are dilution factors of corresponding enzymes; 0, no dilution; 1, 1:5 dilution; 2, 1:25 (5^2^) dilution; 3, 1:125 (5^3^) dilution; 4, 1:625 (5^4^) dilution. Representative gels were shown.

The catalytic defects were further tested for the V256I variant with ensemble relaxation assays using negatively and positively supercoiled plasmid DNA. Both the V256I variant and TOP1MT WT were tested over a dilution range along with nuclear TOP1 used as control. As shown in Fig. 5A, the V256I enzyme was more effective in relaxing negatively- than positively- supercoiled DNA (Sc− vs. Sc+). This preferential activity on Sc− DNA in comparison to Sc+ DNA was observed for all three enzymes tested including nuclear TOP1 (Fig. 5). However, the V256I enzyme was less active than its WT counterpart on both Sc− and Sc+ DNA (compare panels A and B in Fig. 5). These results are consistent with the single-molecule results, indicating that the TOP1MT V256I variant is less effective than the WT TOP1MT in relaxing supercoiled DNA, and positively supercoiled DNA in particular.

### Association of the two TOP1MT variants V256I and R525W with cancer cell lines

We recently analyzed the 60 cancer cell lines of the National Cancer Institute (NCI-60) by whole exome sequencing^17, 22^. Based on the exome sequencing data of the NCI-60 (https://discover.nci.nih.gov/cellminer/), comparison of the six human topoisomerases reveals that TOP1MT has the highest SNP density, with which V256I and R525W being the two most frequent missense variant alleles. The distribution of the two alleles for the lung and melanoma cell lines is presented in Table 3. On one hand, lung cancer cells possess the rs11544484 allele (V256I variant) with much higher frequency than the overall frequency (0.64 vs. 0.27), with 5 out of 9 cell lines homozygous. On the other hand, the rs2293925 allele (R525W) is enriched in melanoma cells (0.65 vs. 0.45 overall frequency), with 6 out of 10 melanoma cell lines homozygous for R525W. Together these results show a bias for each of the two SNPs in melanomas and lung cancer.

**Figure 6:**
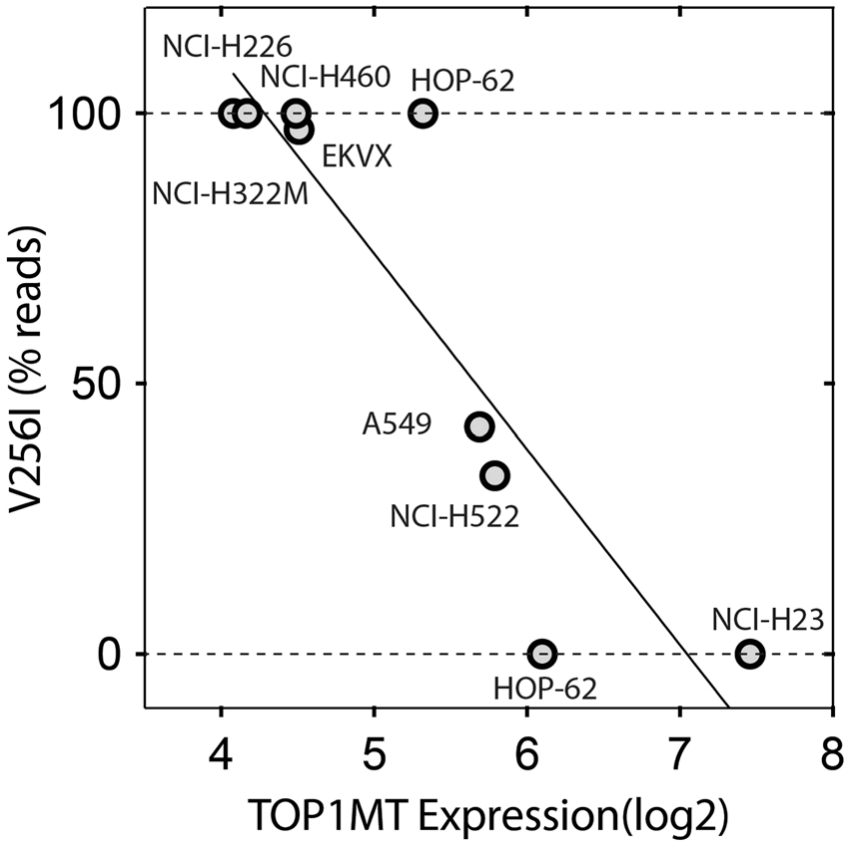
Expression of *TOP1MT* correlates with the V256I SNV in the lung cancer cell lines of the NCI-60.

Another notable finding was that the lung cancer cell lines bearing the V256I variant expressed significantly less *TOP1MT*, with four of the homozygous cell lines (NCI-226, NCI-H322M, NCI-H460 and EKVX) having background (no) *TOP1MT* expression and the two heterozygous cell lines (A549 and NCI-H522) having intermediate *TOP1MT* expression (Fig. 6). These results suggest a negative impact of the *TOP1MT* V256I variant on non-small cell carcinoma cell lines.

## Discussion

Based on genome wide sequencing of the NCI-60 cancer cell lines (https://discover.nci.nih.gov/cellminer/)^17, 22^, we identified the high penetrance (MAF > 0.25) of two human mitochondrial topoisomerase I (TOP1MT) variants: V256I and R525W. A large scale database of human variations was recently released^23^ giving consistent results with our NCI-60 analysis and previously available data from the 1000 genomes (http://browser.1000genomes.org) (Table 1). We evaluated the potential functional impacts ofV256I and R535W using three programs that predict the consequence of mutations on protein function. Both SIFT and Polyphen2 predict that V256I is benign and R525W detrimental, while FATHMM predicts that both V256I and R525W are not deleterious. Consistent with the FATHMM prediction and with the fact that both mutants are present at high frequency in apparently healthy human populations, our biochemical analyses with recombinant enzymes demonstrate that both variants retain robust topoisomerase activity.

SNPs are common genetic variations in human genes with varying frequency. Yet, among the 6 human topoisomerases, only *TOP1MT* possesses variants with a significant MAF, represented by the two SNPs at amino acid positions 256 and 525. There are at least three possible reasons for this phenomenon. First, *TOP1MT* is a nonessential gene^3^, which allows tolerance to mutations and adaptation; second, the *TOP1MT* gene is located at the subtelomeric end of chromosome 8 (8q24.3), an area which is prone to mutations. Third, *TOP1MT* only exists in vertebrates^16^, and thus, from an evolutionary point of view, *TOP1MT* is a relatively new gene, which may still be under selective pressure to optimize the requirements of the nucleus and the mitochondria.

Because most mitochondrial TOP1 (TOP1MT) and all vertebrate nuclear TOP1 enzymes possess an isoleucine at corresponding position 256 of human TOP1MT (Fig. 2 and S1), we surmise that isoleucine was the original amino acid in the ancestral TOP1 serving both in the nucleus and mitochondria, and which is still the case in non-vertebrate eukaryotes^16^. This might explain the highest incidence of the V256I variant in African populations (see Table 1 and Fig. 1B). The human TOP1MT with valine, which presently serves as the reference amino acid in genomic databases might be the result of positive selection in the European and even more in the Asian and Latino populations. An intriguing observation comes from life expectancy of rodents. At the corresponding 256 position, all short living rodents (prairie vole, golden hamster, Chinese hamster, laboratory mouse, rat) have an isoleucine, whereas long life-span rodents (degu, long-tailed chinchilla, domestic guinea pig, naked mole-rat) have a valine.

To better determine the location of the amino acid variants, the structure of TOP1MT could be determined by comparative homology modeling, using the crystal structure of TOP1^24^ as a template. Amino acid 256 is at the junction of two β-sheets, where it is exposed at the surface of the protein (Fig. 1D). In the TOP1 crystal structure, this area corresponds to the “lip” part of core domain 1^5, 15^. Biochemically, we found that the V256I TOP1MT enzyme retains robust topoisomerase activity with only a partial defect for DNA relaxation, especially positively supercoiled DNA (see Figs 3 and 5). The retention of robust catalytic activity is consistent with the high frequency of this variant in the normal human population.

None of the variants except E168G has a direct impact on mitochondrial function despite their reduced catalytic activities. The E168G variant was found in a patient with a mitochondrial deficiency syndrome (65 year-old adult who presented with ptosis, myopathy, neuropathy, type 2 diabetes, calcifications on brain MRI and multiple mtDNA deletions in muscle biopsy; personal communication from Dr. V. Paquis-Fluckinger). It is possible that the mutation affecting negative supercoil relaxation of TOP1MT may be more deleterious on the overall mitochondrial function and proliferation as the E168G variant is the only one to show a defective catalytic activity with negative supercoils. Furthermore, the catalytic deficiencies of the V256I and double mutants V256I-R525W become more apparent and more severe with increasing positive DNA twist density (Fig. 4). The physiological consequences of these defects may therefore be minimized since the high levels of positive supercoiling at which there is a severe loss of activity may not arise frequently, and the single-molecule trajectories suggest that the reduced activity mode is reversible (Fig. 4). From a protein structure and stability standpoint the progressive decrease in catalytic activity with applied torque suggests a torque-dependent structural disruption or rearrangement^12^. This view is consistent with the stochastic switching between catalytically competent and deficient states that favors the deficient state as the positive torque on the DNA is increased. Together these observations are consistent with a model in which torque on the DNA transmitted to the topoisomerase can lead to reversible changes in protein structure or conformation for particular mutants that reduce structural integrity.

Independent of any direct impact on mitochondrial status, it is notable that the 256I variant is highly enriched in the NCI-60 lung cancer lines with 5 of 9 cell lines homozygous for this variant (compare Table 3 and Tables 1 & 2). This suggests a positive selection for cancer cells to bear this variant. Yet, it is also noteworthy that in these same homozygous V256I lung cancer cell lines, expression of the TOP1MT gene is suppressed. This observation suggests a linkage between the V256I variant and lung cancer and a functional impact of the V256I variant in lung cancer cells. Because the I256 residue is on the surface of TOP1MT, it is plausible that it interacts with other proteins in the mitochondria. This might account for the fact that nuclear TOP1, which contains isoleucine as the corresponding residue is toxic to mitochondria^10^, at least in lung cancer cell lines. Therefore, the cells with isoleucine might reduce *TOP1MT* expression to keep its toxicity under control.

All known TOP1MT polypeptides of primates possess arginine (R) at amino acid position 525. At the corresponding position, nuclear TOP1 contains a lysine (K) residue. Based on protein alignments^16^ and on the nuclear TOP1 crystal structure^24^, this arginine is located in the linker region of TOP1MT, an extended α-helix (see Fig. 1)^5, 24^. The mutations in linker region are associated with the topoisomerase activity^25^, resistance to camptothecin^26, 27^, and protein cellular localization^28^. Our biochemical experiments with recombinant R525W TOP1MT demonstrate that this variant has minimal impact on TOP1MT catalytic activity. We interpret the linker region as a modulator of the enzyme activities and contact site of the partners. Some mutations affect enzyme activities and some affect interaction with its partners. Our R525W variant belongs to the later one. It is therefore puzzling why the 525 W variant is expressed with such high penetrance, especially in the Asian populations (see Table 1) and melanoma cancer cell (see Table 3). It is also notable that the penetrance of the 525 W variant is negatively correlated with the presence of the 256I variant in human populations (see Table 1 and Fig. 1B). Namely, Asians and Latinos who preferentially encode the R525W variant tend to have a low frequency for the V256I variant, whereas Africans who preferentially encode the V256I variant have a low prevalence for the R525W variant. This observation suggests negative selection against the double TOP1MT variant 256I and 525 W in human populations. This is even more obvious in our genotyping analysis of 129 human samples (see Table 2), which shows mutual exclusion between homozygocity for one of the alleles and presence of the second allele. The same is apparent when comparing the NCI-60 lung cancer and melanoma cell lines (see Table 2 and Supplemental Table 1). Each tissue type shows a strong bias for one variant (V256I for non-small cell lung cancer cells and R525W for melanoma) and excludes the other. Yet, this cannot be readily explained on the basis of our biochemical analyses as the recombinant protein with both variants was functionally competent.

Together, our results demonstrate the value of biochemical experiments to evaluate the functional importance of genomic variants but also the limitations of biochemical tests. Based on the selection of each of the variants in human populations and cancer cell lines, as well as their mutual exclusion, it is likely that the functional importance of these variants is determined in the context of the mitochondrial environment, possibly by protein-protein interactions or macromolecular complexes that regulate TOP1MT functions. Further studies are warranted to determine the potential linkage of the two SNP variants (V256I and R525W) in lung cancers and melanomas and to determine the possible relevance of TOP1MT mutations as a source of mitochondrial diseases and susceptibility to therapies^8^.

## Materials and Methods

### Drug, enzymes, and supercoiled DNA

Camptothecin (CPT) was provided by the Developmental Therapeutics Program (DTP), DCTD, NCI, NIH (Bethesda, MD). Human nuclear TOP1 and TOP1MT were purified from baculovirus as previously described^12^. Plasmid pBR322 (NEB, Ipswich, MA) was used as (−) supercoiled substrates, whereas (+) supercoiled substrates were generated by incubating negative supercoiled pBR322 with *Archaeoglobus fulgidus* reverse gyrase following the protocol of McClendon *et al*.^13^ and modified according to Hsieh and Capp^29^ to make highly positively supercoiled DNA using an enzyme to DNA mole ratio of 40:1.

### Genotyping analyses for the V256I and R525W variants in human samples

Allelic discrimination was used to genotype *TOP1MT*. The assays of allelic discrimination for V256I (variant ID: rs11544484) and R525W (variant ID: rs2293925) were carried out on the Applied Biosystems 7900HT fast real-time PCR system. The allelic discrimination primer/probe sets for rs115444484 and rs2293925 were purchased from Thermo Fisher. Following the manufacturer’s protocol, the allelic discrimination assay classifies samples as homozygotes and heterozygotes.

### Single molecule DNA supercoil relaxation assay

“Coilable” 11 kb DNA molecules were used as substrates for the single-molecule DNA supercoil relaxation assay employing a custom-built magnetic tweezers instrument as previously described^11, 12, 20^. Briefly, the 11 kb DNA tether multiply labeled with biotin on one end and digoxigenin on the other was attached to an anti-digoxigenin coated surface and a 1 µm streptavidin magnetic particle. DNA supercoils were generated by rotating external magnets, which decreased the extension (height) of the bead as the linear DNA was transformed into plectonemes (Fig. 4A). During the assay, supercoil relaxation activity by TOP1MT was measured by tracking the height of the bead at 200 Hz using video tracking routines as described previously^12^. The DNA extension increases as TOP1MT removes supercoils. Relaxation rates were quantified by a linear fit over the region where DNA extension increases whereas the uncoiling step sizes (number of turns relaxed per cleavage-religation cycle) were estimated by measuring the DNA extension change during the burst of relaxation (Fig. 4A) using custom written software in Igor Pro (Wavemetrics)^12^. Supercoil relaxation measurements were performed in topoisomerase buffer (10 mM Tris pH 8, 50 mM KCl, 10 mM MgCl_2_, 0.3% w/v BSA, 0.04% Tween-20, 0.1 mM EDTA, and 5 mM DTT) with 0.1**–10** nM of TOP1MT.

### Assays for TOP1- and TOP1MT-mediated relaxation of plasmid *DNA*

Relaxation reactions were carried out in reaction buffer [10 mM Tris-HCl, pH 8.5, 50 mM KCl, 5 mM MgCl_2_, 0.1 mM EDTA, 5 mM DTT, and 15 μg/mL BSA]. A total of 200 fmol of negatively or positively supercoiled pBR322 were incubated with TOP1 or TOP1MT enzyme at 37 °C for 30 minutes and stopped by addition of SDS to a final concentration of 0.2% (w/v). After digestion with 0.5 µg/ml of proteinase K at 37 °C for 30 minutes, the products were separated in 1% agarose gels. The gels were stained with 0.5 μg/ml ethidium bromide to visualize DNA.

### TOP1 cleavage assay with a linear fragment of pSK DNA

The DNA cleavage protocol was carried out as reported^30^. Briefly, a 118-bp DNA oligonucleotide was generated from a 117/118-bp oligonucleotide of pSK DNA containing a single 5’-cytosine overhang, which was 3’-end-labeled by fill-in reaction with [^32^P]dGTP with 0.5 units of DNA polymerase I (Klenow fragment, New England BioLabs). Unincorporated [^32^P] dGTP was removed using mini Quick Spin DNA columns (Roche, Indianapolis, IN), and the eluate containing the 3’-end-labeled DNA substrate was collected. Approximately 2 nM of radiolabeled DNA substrate was incubated with recombinant TOP1MT or TOP1 in 10 μL of reaction buffer [10 mM Tris-HCl, pH 8.5, 50 mM KCl, 5 mM MgCl_2_, 0.1 mM EDTA, 5 mM DTT and 15 μg/mL BSA] at 25 °C for 20 min. Reactions were stopped with SDS (0.5% final concentration) followed by the addition of equal volumes of loading dye (80% formamide, 10 mM sodium hydroxide, 1 mM sodium EDTA, 0.1% xylene cyanol, and 0.1% bromphenol blue). Aliquots of each reaction mixture were subjected to 20% denaturing PAGE. Gels were dried and visualized using a phosphoimager and ImageQuant software (GE Healthcare Bioscience Corp, Piscataway, NJ).

## Electronic supplementary material

Supplementary Figures S1 and S2
