# Supplementary material for: Distribution bias and biochemical characterization of TOP1MT single nucleotide variants
Source: Sci Rep. 2017 Aug 17;7:8614. doi: 10.1038/s41598-017-09258-2 (PMC5561071; doi:10.1038/s41598-017-09258-2)
Supplement: Supplementary file 1 — Supplementary Figures S1 and S2 [file 41598_2017_9258_MOESM1_ESM.pdf]

Distribution bias and biochemical characterization of *TOP1MT* single nucleotide variants

Hongliang Zhang[1], Yeonee Seol[2], Keli Agama[1], Keir C. Neuman[2], and Yves Pommier[1*]

[1]Laboratory of Molecular Pharmacology, Developmental Therapeutics Branch, Center for Cancer Research, NCI, National Institutes of Health, Bethesda, MD, 20892, USA

[2] Laboratory of Single Molecule Biophysics, NHLBI, National Institutes of Health, Bethesda, MD, 20892, USA

[*]For correspondance: pommier@nih.gov

Formatted Alignments

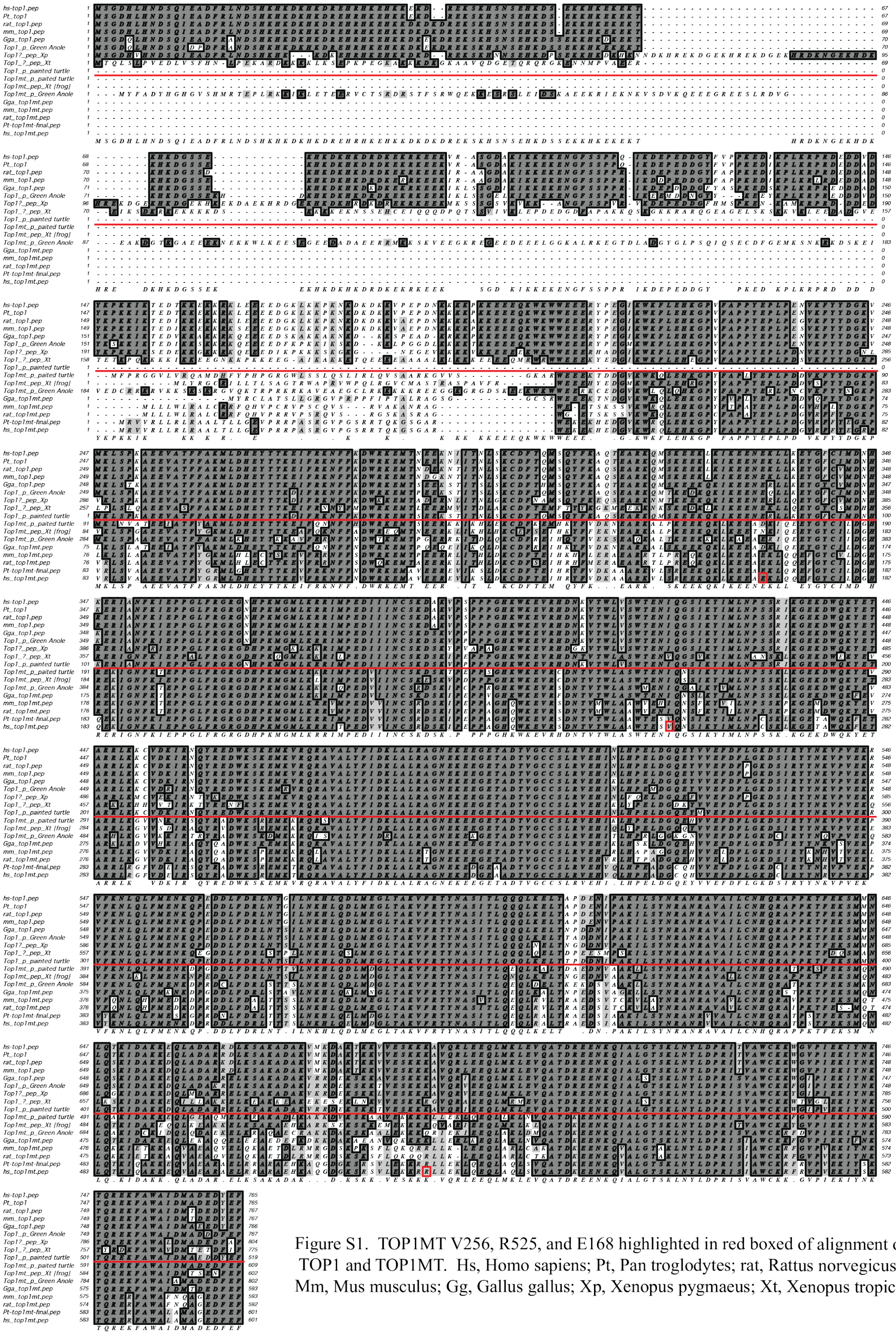

Figure S1. TOP1MT V256, R525, and E168 highlighted in red boxed of alignment of TOP1 and TOP1MT. Hs, Homo sapiens; Pt, Pan troglodytes; rat, Rattus norvegicus; Mm, Mus musculus; Gg, Gallus gallus; Xp, Xenopus pygmaeus; Xt, Xenopus tropicalis.

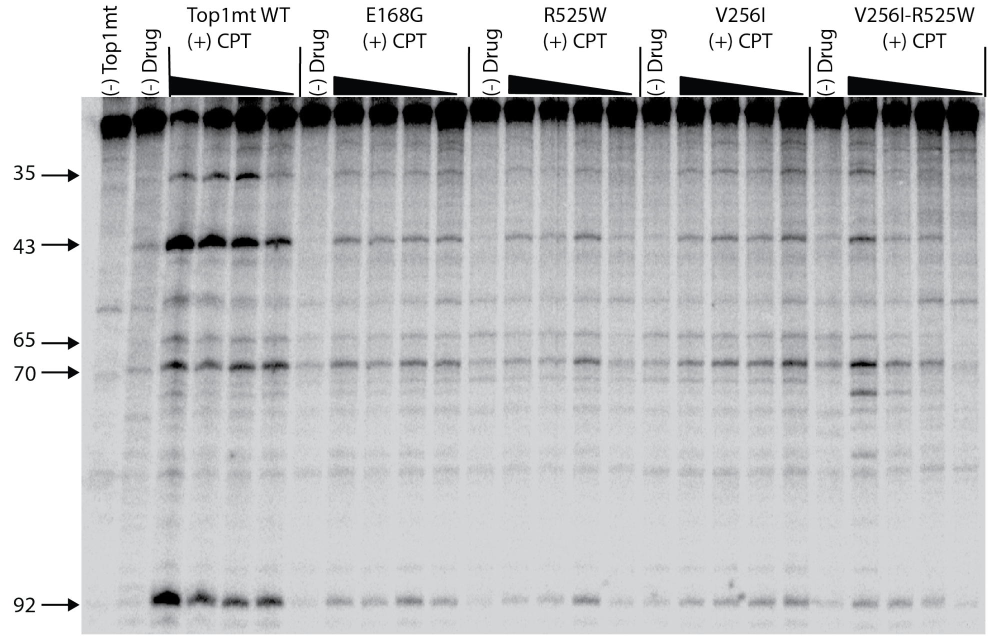

Figure S2. Cleavage assay of purified Top1mt mutant proteins: (lane 1) DNA alone; (lane 2) Top1mt alone; (lane 3-6) 1:3 fold serial dilution of Top1mt incubated with 1 µM camptothecin; (lane 7) E168G Top1mt alone; (lanes 8-11) 1:3 fold serial dilution of E168G Top1mt incubated with 1 µM camptothecin; (lane 12) R525W Top1mt alone; (lane 13-16) 1:3 fold serial dilution of R525W Top1mt incubated with 1 µM camptothecin; (lane 17) V256I Top1mt alone; (lane 18-21) 1:3 fold serial dilution of V256I Top1mt incubated with 1 µM camptothecin; (lane 22) R525W-V256I Top1mt alone; (lane 23-26)) 1:3 fold serial dilution of R525W-V256I Top1mt incubated with 1 µM camptothecin.
